#  The POLG Polyglutamine Tract Variants in Iranian Patients with Multiple Sclerosis 

**Published:** 2015

**Authors:** Mehri KHATAMI, Mohammad Mehdi HEIDARI, Reza MANSOURI, Fatemeh MOUSAVI

**Affiliations:** 1 Department of Biology, Faculty of Science, Yazd University, Yazd, Iran; 2 Department of Immunology, Shahid Sadoughi University of Medical Science, Yazd, Iran

**Keywords:** Multiple sclerosis, POLG gene, CAG repeats, Trinucleotide expansion

## Abstract

**Objective:**

Multiple Sclerosis (MS) is a common disease of the central nervous system. The interaction between inflammatory and neurodegenerative processes typically results in irregular neurological disturbances followed by progressive disability. Mitochondrial dysfunction has been implicated in neurodegenerative disorders. The DNA polymerase-gamma (POLG) gene, which encodes the catalytic subunit of enzyme responsible for directing mtDNA replication, contains a poly glutamine tract (poly-Q) in the N-terminal, encoded by a CAG sequence in exon 2.

**Materials & Methods:**

We analyzed the POLG trinucleotide repeats in 40 Iranian patients with MS (27 females and 13 males with an age range of 18–55); and 47 healthy age, gender, and ethnic matched controls were chosen by PCR-SSCP analysis.

**Results:**

Our results indicated that the most common allele in patients had 10 consecutive CAG repeats (10Q). Other alleles of 11and 12 trinucleotide repeats were detected. We did not find any difference between the CAG repeat length distribution in controls and MS patients.

**Conclusion:**

No correlation was observed in the POLG gene CAG repeat with pathogenesis of MS, but it looks that other point mutations in POLG gene may have an important role in the disease’s pathogenesis and produced more significant results.

## Introduction

Multiple Sclerosis (MS) is a chronic and a common inflammatory disorder of the central nervous system (CNS) characterized with myelin loss, progressive neurological dysfunction, gliosis, and unstable degrees of axonal pathology (1). Prevalence rates for MS vary between 2 and 2.5 million individuals worldwide(2). While extensive investigation has been performed on the etiology and the pathogenesis of MS, up to now, no specific treatment, diagnostic test, or reliable biomarker has yet been recognized for MS patients(3). 

Epidemiological studies in the relatives of affected individuals have shown that genetic risk factors are mainly responsible for a considerably increased frequency of the disease (4). Recently, other research suggests that disorders of the mitochondrial were present in patients with MS(5, 6). 

The only known mitochondrial DNA (mtDNA) polymerase is DNA polymerase γ (POLG) (7). The integrity of mtDNA is maintained by POLG(8). Its mature form contains of a polymerase domain with three polymerase motifs: A, B, and C; and an exonuclease domain. The POLG exonuclease domain consists of a CAG repeat region (encoded poly-Q) in exon 2, which is responsible for proofreading activity of the encoded enzymes(9). The length variation of the poly-Q may modulate enzyme function(10). Deletion analysis of the CAG repeat regions has shown that it may not effect enzymatic properties, but reasonably up-regulates the expression(11, 12). This CAG repeat explanation underlies various neurodegenerative disorders(13). CAG Alternations have been found to be associated with idiopathic sporadic Parkinson’s(14), Friedreich ataxia(15), and Varicocele(16). The purpose of this study was to evaluate the association of POLG CAG repeat length with MS risk. 

## Materials & Methods

**Patients**

Relevant information was obtained from 40 patients with definite MS of all subtypes (Table 1). All study subjects gave informed consent. Further, 47 ethnically matched healthy unrelated individuals were enrolled into the study as the control group. All patients and the control group were informed of the aims of the study as well as gave informed consent for genetic analysis. Patients were diagnosed and referred for assessment by consultant neurologists from Yazd, Iran.

**Table 1:** Clinical Characteristics of The MS Patients

	**Relapsing-remitting** **(** ***n*** ** = 31)** a	**Secondary progressive** **(** ***n*** ** = 4)** b	**Progressive remitting** **(** ***n*** ** = 3)** c	**Primary progressive** **(** ***n*** ** = 2)** d
**Age**	32.54 ± 8.51	36 ± 11.91	32± 8.7	50 ± 7.07
**Age of onset (year)**	28.61 ± 8.42	33.5 ± 12.26	30.3 ± 9.4	39.5 ± 0.7

a : 18 female and13 male-

b, c, d : female

**DNA analysis**

Genomic DNA was extracted from peripheral blood samples using a salting out technique. 286-bp DNA fragment were amplified by PCR in a volume of 25 μl, using two primers (GGTCCCTGCACCAACCATGA and CTTGCCCGAAGATTTGCTCGT); it has been published previously (17). 

We used the SSCP technique for mutation analysis of the POLG gene. For the SSCP assay, PCR products were heat-denatured at 93ºC for 3 min and chilled on ice for 3 min, and loaded onto a polyacrylamide/TBE 0.5x gel. After the run, the gel was removed from the apparatus and the DNA bands were visualized through silver staining. The SSCP variant bands on the gel were sent to be sequenced by an automated sequencing 3700 ABI machine (Macrogene Seoul, South Korea). 

**Statistical Method**

The GraphPad Prism software was used for statistical analysis. The Fisher’s exact test was used to assess the association POLG CAG repeats in MS patients and the control group. P-values of <0.05 were considered statistically significant. 

## Results

Table 2 shows the allele and genotype frequencies of the CAG polymorphism in patients with MS and controls. In both patients and controls, there was a strong predominance of the allele 10Q. We did not find a statistically significant difference between the two studied groups. The frequency of the common POLG variant among the MS patients was 92.5%, which was similar to the control group (85.1%), (p=0.155). CAG repeat lengths ranging from 10–12 were detected, but not larger, expanded repeats were found in the control subjects and MS patients.

**Fig 1:**
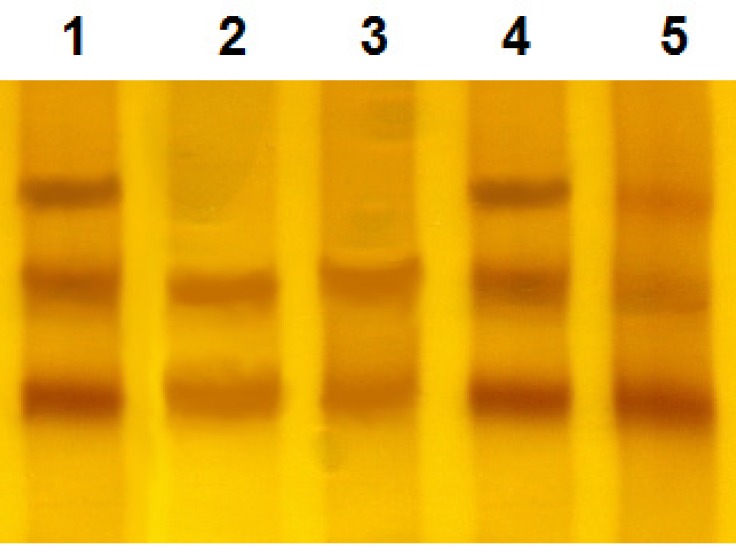
Genotyping of POLG CAG repeat by PCR-SSCP. 1, 4, and 5 are 10/≠10 genotype and 2, 3 are 10/10 genotype

**Table 2:** POLG Exon 2 Poly Q (CAG-Repeat) Allele Frequencies

Allele(number of CAG repeat)	No. of alleles in patients	No. of alleles in controls	*P*–value	Odd ratio
10	74(92.5%)	80 (85.1%)	0.155	2.15
11	5 (6.25%)	13 (13.82)	0.1349	0.415
12	1 (1.25%)	1 (1.06%)	1.000	1.17
Total	80	94	-	
Genotype	No. of patients	No. of controls		
10/10 homozygous	34 (85%)	33 (70.2%)	0.128	2.40
10/≠10 heterozygous	6 (15%)	14 (29.8%)	0.065	0.4160
	40	47	-	

## Discussion

DNA polymerase gamma is the only DNA polymerase active in the mitochondria. Interestingly, the human POLG gene includes a trinucleotide CAG repeat that is absent from the orthologue in other metazoans and yeast. The microsatellite repeat encodes a polyglutamine tract in the amino-terminal region of the POLG protein, downstream of the presumed mitochondrial targeting sequence(18).Mutations in the POLG gene have emerged as one of the most common causes of inherited mitochondrial disease in children and adults. They are responsible for a heterogeneous group of major phenotypes of neurodegenerative disease that include: childhood Myocerebrohepatopathy Spectrum disorders (MCHS) and Alpers-Huttenlocher syndrome, as well as mtDNA deletion disorders in the Ataxia Neuropathy Spectrum (ANS), which includes spinocerebellar ataxia with epilepsy (SCAE) and mitochondrial recessive ataxia syndrome without ophthalmoplegia (MIRAS); Myoclonus Epilepsy Myopathy Sensory Ataxia (MEMSA); and autosomal dominant and recessive forms of progressive external ophthalmoplegia (PEO), which may include sensory ataxic neuropathy with dysarthria and ophthalmoparesis (SANDO), and cases of parkinsonism in PEO+ (19). Many studies show the involvement of the mitochondria in MS. First, ultrastructural analysis of demyelinated spinal cord lesions showed dramatically reduced numbers of mitochondria(5). Second, mitochondrial protein or DNA altered by mutation may trigger pathological immune response against self-epitopes potentially contributing to an autoimmune process(20). Third, a potential role for inherited mtDNA defects in MS is suggested by the tendency towards maternal inheritance, a hallmark of primary mitochondrial disorders, in a parent of origin half sibling study(21).Fourth, a number of MS cases bearing pathogenic mtDNA defects with Leber’s hereditary optic neuropathy (LHON) and MS (Harding’s disease) being the most well recognized, has been reported(22). Fifth, mutations in UCP2 (866 G/A), ND2(5)and ATPase6(23)genes are reported to be implicated in patients with MS. Sixth, an energy deficient state has been implicated in the generation of axons the pathological correlate of disease progression in MS(24).The allele distribution of the poly-Q repeat of the POLG is relatively uniform in humans, as over 80% of people in all studied populations carry the 10Q allele(11, 14, 15). Natural selection is a factor that could explain this, but is not clear at present whether purifying selection or low mutation rate is responsible for the unusually uniform allele distribution(10). Analysis CAG repeat length in Iranian MS patients samples did not show any detectable and no notable difference from that in the control subjects. The data presented in this study are inconsistent with the pathogenic role of the CAG polymorphism in MS. 

Our previous findings on Friedreich’s ataxia patients showed a statistically significant inverse correlation between the POLG CAG repeats and age of onset in FRDA patients(15). Taanmana et al. has identified that the POLG CAG repeats are not a contributory factor in idiopathic Parkinson’s disease(18). These results warrant further study and more samples are needed to produce more significant results. 

## Notes

**Financial Disclosures: **None declared.

## Notes

**Funding/Support: **None declared.
